# The Effect of Stromal Integrin β3-Deficiency on Two Different Tumors in Mice

**DOI:** 10.3390/cancers8010014

**Published:** 2016-01-12

**Authors:** Inga Reigstad, Kristina Sortland, Trude Skogstrand, Rolf K. Reed, Linda Stuhr

**Affiliations:** 1 Department of Biomedicine, University of Bergen, Jonas Lies vei 91, N-5009 Bergen, Norway; kristina.sortland@uib.no (K.S.); trude.skogstrand@uib.no (T.S.); linda.stuhr@uib.no (L.S.); rolf.reed@uib.no (R.K.R.); 2 Matrix Biology Group, Haukeland University Hospital, N-5021 Bergen, Norway; 3 Center of Cancer Biomarkers, University of Bergen, N-5020 Bergen, Norway

**Keywords:** angiogenesis, fibrosis, interstitial fluid pressure, metastasis, tumor growth

## Abstract

There is an increasing focus on the tumor microenvironment in carcinogenesis. Integrins are important receptors and adhesion molecules in this environment and have been shown to be involved in cell adhesion, proliferation, differentiation and migration. The present study aimed to evaluate the effect of stromal integrin β3-deficiency on tumor growth, angiogenesis, interstitial fluid pressure (PIF), fibrosis and metastasis in a murine breast cancer (4T1) and a prostate tumor (RM11) model. We showed that stromal integrin β3-deficiency led to an elevation in PIF that correlated to a shift towards thicker collagen fibrils in the 4T1 mammary tumor. In the RM11 prostate carcinoma model there was no effect of integrin β3-deficiency on PIF and collagen fibril thickness. These findings support the notion that changes in the collagen scaffold influence PIF, and also indicate that there must be important crosstalk between the stroma and tumor cells, in a tumor cell line specific manner. Furthermore, stromal integrin β3-deficiency had no effect on tumor growth or angiogenesis in both tumor models and no effect on lung metastasis in the 4T1 mammary tumor model. In conclusion, the stromal β3 integrin influence PIF, possibly via its effect on the structure of the collagen network, in a tumor cell line dependent manner.

## 1. Introduction

For many years the primary focus and target in cancer research has been on tumor cells. Lately, however, there has been an increasing focus on the role of the tumor microenvironment in carcinogenesis. The tumor microenvironment, as other normal tissues of the body, contains endothelial cells, pericytes, immune cells, fibroblasts and extracellular matrix (ECM). All these components are important contributors to carcinogenesis [1,2].

Disorganization of the tissue architecture is characteristic for tumors. The ECM is characterized by increased fibrosis, and thus tumor stiffness [2,3]. Furthermore, the blood vessels in the tumor microenvironment are dysfunctional and leaky, and the lymph vessels are defective or absent. These factors are suggested to contribute to the increased interstitial fluid pressure (PIF) found in most solid tumors [4]. According to the Starling hypothesis, an enhanced PIF can act as a functional barrier and lead to impaired uptake of anticancer drugs into the tumors [4].

Integrins are heterodimeric membrane receptors that sense and integrate the information from ECM proteins, cytokines, growth factors, immunoglobulins and matrix degrading proteases [5]. They mediate information between cells and the extracellular matrix, and between cells, both outside-in and inside-out, affecting cell adhesion, proliferation, cell survival, differentiation and migration [6].

The combination of the 18α- and 8β–subunits defines the ligand specificity and signaling properties of the particular integrin [5]. The present study has focused on the β3 integrin subfamily. The β3 integrin subfamily consists of αIIbβ3 and αvβ3. While integrin αIIbβ3 is mainly expressed on platelets and megakaryocytes [7], integrin αvβ3 is normally expressed on many cell types including endothelial cells, smooth muscle cells, fibroblasts, monocytes, osteoclasts as well as platelets [7,8]. Integrin αvβ3 is found in adult epithelia and can be highly upregulated in certain tumor cells [7,9]. The integrin αvβ3 is upregulated in tumor-associated blood vessels, and has been proposed to be involved in regulation of angiogenesis [10]. The αvβ3 integrin associates with PDGF-Rβ and VEGF-R2, and these receptors play a role in cell survival and proliferation [11,12]. The expression of β3 integrins is also associated with the ability of tumors to metastasize [7,13,14], and some preclinical studies have shown that metastases can be reduced by integrin αvβ3-antagonists [15,16,17,18,19].

Thus, the αvβ3 integrin has been implicated in both tumor progression and metastasis, but with variable, and even contradictory results, in both experimental as well as clinical studies using αvβ3-antagonists [20,21,22,23,24,25,26,27,28,29,30].

Mice lacking stromal integrin expression can provide functional insight into the specific integrin of choice and we therefore chose an integrin β3 knockout mouse model to study the function of the β3 integrin in carcinogenesis. The specific aim of the present study was to investigate the effect of *stromal* integrin β3-deficiency on tumor growth, angiogenesis, interstitial fluid pressure, fibrosis and metastasis, in two different types of allografted murine carcinomas, the 4T1 metastatic breast and the RM11 prostate carcinoma.

## 2. Methods

### 2.1. Cell Lines

The murine mammary carcinoma cell line 4T1 was obtained from the American Type Culture Collection (Rockville, MS, USA). The prostate cell line RM11 was a kind gift from Associate Professor Thomas S. Griffith (University of Minnesota, Minneapolis, MN, USA). The cells were grown in RPMI-1640 medium (HEPES solution for RM11 cells) supplemented with 10% Fetal Bovine Serum (Sigma-Aldrich, Steinheim, Germany), 100 units/mL penicillin, 100 μg/mL streptomycin, 1%–2% l-glutamine (all from Bio-Whittaker, Walkersville, MD, USA), with an addition of 1% sodium pyruvate for the RM11 cells. All cells were grown as a monolayer in a humidified incubator at 37 °C, in 5% CO_2_ and 95% air, and were seeded and used at log phase in all experiments.

### 2.2. Animal Model

The BALB/c integrin β3-deficient (β3-KO) and wild type (WT) mouse strains was originally gifts from Professor Kristofer Rubin (Uppsala University, Uppsala, Sweden). Female mice were used for the mammary 4T1 model, and male mice for the prostate RM11 model. The animal experiments were performed in accordance with the regulations of the Norwegian Animal Research Authority and approved by the local ethical committee (project number 20124127).

### 2.3. Establishing Primary Tumors

A total of 3 × 10^5^ 4T1 tumor cells in 0.15 mL PBS were injected into the mammary fat pads on each side of the groin area. In the prostate tumor study, 2 × 10^5^ RM11 cells were injected subcutaneously on both sides of the mouse flank. The 4T1 tumors were measured using a caliper on days 7, 10, 13 and 17, and RM11 tumors on days 14, 17 and 20 post-injection. The tumor volume was calculated using the formula; *tumor volume (mm^3^) = (π/6) × a^2^ × b*, where *a* represents the shortest diameter of the tumor and *b* represents the longest diameter of the tumor. All animals were anesthetized by isoflurane (Isoba®vet. 100%, Schering-Plough A/S, Farum, Denmark) in combination with N_2_O and O_2_ during experiments. The animals were sacrificed during anesthesia. The experiments ended day 17 post-injection for the 4T1 tumors and day 20–21 for the RM11 tumors. The metastases study was performed separately and is described below.

### 2.4. Interstitial Fluid Pressure

The tumor interstitial fluid pressure (PIF) was measured using the wick-in-needle technique (WIN) [31]. Briefly, a standard 23-gauge needle with a side hole, filled with nylon floss and saline, was inserted into the central part of the tumor and connected to a PE-50 catheter, a pressure transducer and a computer for pressure registrations. After a period of stable pressure measurements, the fluid communication was tested by clamping the catheter which should cause a transient rise and fall in pressure. Measurements were accepted if the pre to post-clamping value was within ± 1 mmHg. The PIF-measurements were performed on the last day of the experiment.

### 2.5. Electron Microscopy of Collagen Fibrils in the Tumor

A JEM-1230 Transmission Electron Microscope (TEM),(Jeol, Tokyo, Japan) was used to measure the diameter of the collagen fibrils. The tissue samples were cut into approximately 1 × 1 × 1 mm samples and fixed in 2.5% glutaraldehyde in 0.1 M phosphate buffer, and then washed in PBS. The samples were post-fixed in 1% OsO_4_ in PBS and dehydrated in increasing concentrations of 70%, 95% and 100% ethanol, and then propylenoxide, before being embedded in Agar 100 Resin and sectioned at 60 nm. One section was used per tumor. At least five images from different areas of the tumors, and 3–6 images from different areas of dermis, were captured at a magnification ×100,000 and analyzed using Image J 1.46 (National Institutes of Health, Beteshda, MD, USA). Because of uneven distribution of collagen in the tissue, the images were taken from the areas of the tissue where collagen was found.

A Jeol JSM-7400F Scanning Electron microscope (SEM) was used to study the tumor collagen scaffold architecture. The tumors were cut in 1 × 1 × 1 mm samples and fixed in 2.5% glutaraldehyde in 0.1 M phosphate buffer, before being placed in 10% NaOH for 7 days with replacement every day. The NaOH was then replaced with tap-water for 2–4 days and dehydrated in increasing concentrations of 70%, 95% and 100% ethanol, and dried in a “critical point-dryer”. The tumor tissue was mounted on an Au-stub and coated with a 10 nm layer of gold and palladium using a Jeol JFC-2300HR High Resolution fine coater. Five images from different areas of the tumor were captured from each tumor at a magnification ×10,000.

### 2.6. Immunohistochemistry and Immunofluorescence

Frozen 10 µm tumor sections were used for immunohistochemistry and immunofluorescence. One section was used per tumor. To visualize tumor blood vessels, CD31 staining, a two-step indirect method was used. Rat anti-mouse CD31 (dilution 1:200, AbD serotec, Morphosys UK Ltd., Oxford, UK) was used as primary antibody and biotinylated rabbit anti-rat (dilution 1:200, Vectastain ABC kit, peroxidase Rat IgG PK 4004, Vectors Laboratories, Inc., Burlingame, CA, USA) as secondary antibody.

Ki67 staining was used to visualize cell proliferation. Prior to Ki67 staining, the sections were placed in an antigen retrieval solution made of citrate buffer for 25 min at 97 °C. A two-step indirect method was used. Rat anti-mouse Ki67 antigen (M7249, clone TEC3, dilution 1:100, Dakocymation, Denmark A/S, Glostrup, Denmark) was used as primary antibody and biotinylated goat anti-rat (E0468, dilution 1:100, Dakocymation, Denmark A/S) as secondary antibody. 3,3’-Diaminobenzidine tetrahydrochloride (Sigma-Aldrich) was used as a chromogen for both these immunostaining protocols, and Richardson stain was used as counterstain.

In an organized pattern, representative images from 4T1 and RM11 tumors were captured with a Nikon camera (Nikon Digital Sight, Nikon Corporation, Tokyo, Japan) at ×10 magnification. Five images were captured from each 4T1 tumor and 1–3 images from each RM11 tumor, as these were smaller in size. The average number of blood vessels (vessel/mm^2^) or proliferating cells (% of total cells) was calculated.

For α-SMA staining, monoclonal anti-actin α-smooth muscle–FITC-conjugated antibody (F3777, dilution 1:200/1:300, Sigma-Aldrich) was used. For NG2 staining, rabbit anti-NG2 chondroitin sulfate proteoglycan (AB5320, dilution 1:100, Merck Millipore, Darmstadt, Germany) was used as primary antibody and goat anti-rabbit IgG, Alexa Fluor 488 conjugate (A-11034, dilution 1:300, Life Technologies, Thermo Fisher Scientific, Waltham, MA, USA)/goat anti-rabbit IgG, Alexa Fluor 594 conjugate (111-585-144, dilution 1:300, Jackson Immunoresearch Laboratories, Inc., West Grove, PA, USA) as secondary antibody.

Five representative images from each 4T1 tumor at ×20 magnification were acquired with an Axioscope fluorescence microscope and a digital Axiocam MRm camera (Zeiss, Oberkochen, Germany). To identify the amount of pixels positive for α-SMA and NG2, Fiji ×64 (National Institutes of Health, Beteshda, MD, USA) was used. An individual threshold value was used for each picture to adjust for background.

### 2.7. Metastasis

To allow for development of metastasis, female animals were injected with 5 × 10^5^ 4T1 cells in one mammary fat pad. The primary tumors were resected on day 15 or 16 post-injection due to their size, and the surgical wound was closed by tissue glue. The experiment was terminated on day 27 post-injection.

The liver and femur bone were removed and fixed in formalin immediately after sacrificing the animal. After fixation the bone was decalcified in 10% EDTA, pH 7.2 during a period of 5 weeks. The lungs were fixed using approximately 1 mL of Bouin’s solution (Gurr BDH Chemicals Ltd., Poole, UK) injected into the trachea. The lungs were immediately dissected out, fixated in new Bouin’s solution, washed in 70% ethanol, dehydrated and embedded in paraffin using standard procedures. Sections were stained with H & E staining and examined by light microscopy.

In order to quantify lung metastases, 4 coronal sections from both lungs from each animal were examined. From one of the animals only 1 lung was analyzed due to a total collapse of the second lung. Total number of metastases per lung was counted, and the area per lung covered by metastases was measured in mm^2^ (Nikon Digital Sight, Nikon Corporation).

### 2.8. Statistical Methods

For statistical analysis, Sigmaplot 12.5 (Systat Software Inc., Chicago, IL, USA ) was used. Either the unpaired two-tailed *t*-test, or the Mann-Whitney rank sum test, was used to analyze statistical differences between the two groups. Results were accepted as statistically different when *p* < 0.05 in two-tailed testing. Graph Pad Prism 6 (GraphPad Software, Inc., La Jolla, CA, USA) was used to create all figures. Data is given as mean ± SD, and number of measurements (n) refers to number of tumors unless otherwise specified.

## 3. Results

### 3.1. Stromal Integrin β3-Deficiency and Tumor Growth

To evaluate the effect of stromal integrin β3 on tumor growth, 4T1 mammary tumor cells and RM11 prostate tumor cells were injected in BALB/c integrin β3 wild type (WT) and integrin β3-deficient (β3-KO) mice, and tumor volumes were measured at different time points using a caliper.

**Figure 1:**
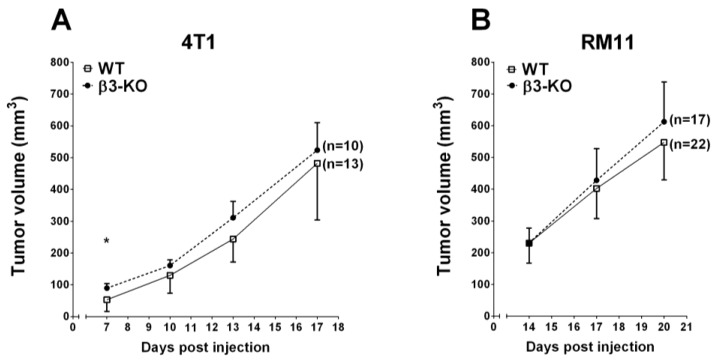
The growth of 4T1 (**A**) and RM11 (**B**) tumors in WT and β3-KO mice measured every other third day. A total of 3 × 10^5^ 4T1 and 2 × 10^5^ RM11 cells were injected into the fat pad and subcutaneously on the mouse flank, respectively. Mean ± SEM. * *p* < 0.05.

At day 7, post-injection, the 4T1 tumor volume in β3-KO mice was significantly larger (89.5 ±14.2 (SEM) mm^3^, *n* = 10) than in WT mice (52.9 ± 10.2 (SEM) mm^3^, *n* = 13). However, at days 10, 13 and 17 post-injection, there were no significant differences in tumor volume between the β3-KO mice and WT mice (Figure 1A). Furthermore, there were no significant differences in RM11 tumor volume between the β3-KO mice (*n* = 17) and WT mice (*n* = 22) on days 14, 17 and 20 post-injection (Figure 1B). Thus, stromal integrin β3-deficiency did not influence tumor growth in 4T1 or RM11 allografts during the later stages of tumor progression.

### 3.2. Integrin β3-Deficiency in Stromal Cells Has No Effect on Blood Vessels, α-SMA or Cell Proliferation

**Figure 2:**
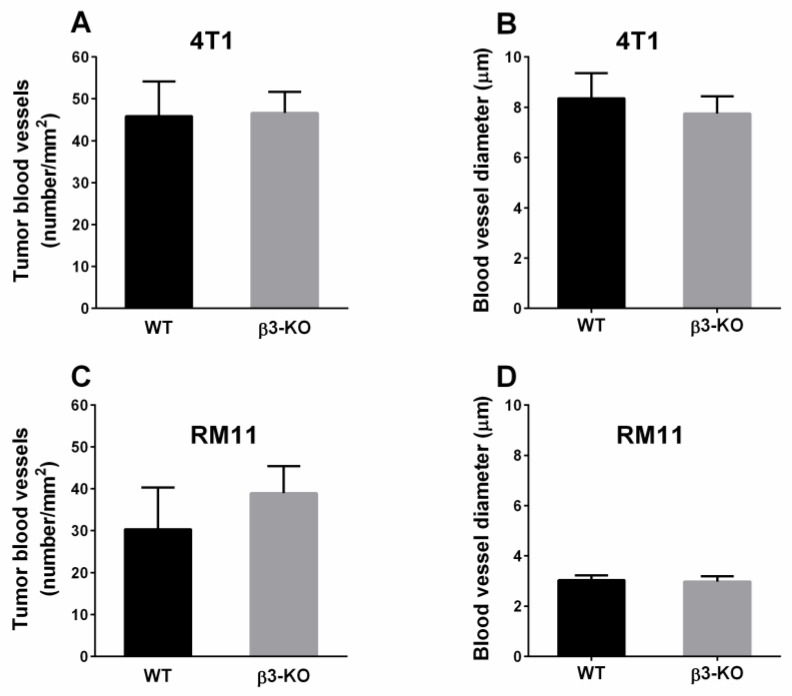
Microvascular density (**A**,**C**) and diameter (**B**,**D**) in orthotopic 4T1 (*n* = 4 and *n* = 5) and subcutaneous RM11 (*n* = 5) tumors were calculated using immunohistochemical detection of CD31. No statistical differences in tumor blood vessel density (4T1 *p* = 0.86, RM11 *p* = 0.14) or diameter (4T1 *p* = 0.36, RM11 *p* = 0.69) were found. Mean ± SD.

**Figure 3:**
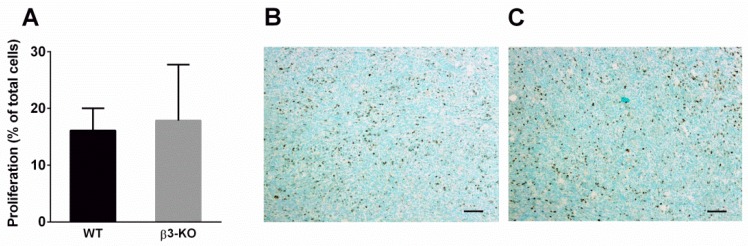
Percentage of proliferating cells of total cells was calculated using immunohistochemical detection of Ki67 in 4T1 (*n =* 4) tumors (**A**) obtained from WT and β3-KO mice. No statistical difference was found (*p* = 0.76). Mean ± SD. Representative images from both genotypes are shown (**B**,**C**). Scale bars indicate 100 µm.

It has previously been indicated that angiogenesis is influenced by stromal integrin αvβ3 [10]. CD31-immunostaining showed a large number of blood vessels in the tumor sections. However, there were no significant differences in blood vessel density in neither 4T1 nor RM11 carcinomas between β3-KO mice (*n* = 5) and WT mice (*n* = 5) (Figure 2A,C). Furthermore, there were no significant differences in blood vessel diameter in 4T1 (*n* = 4) and RM11 (*n* = 5) tumors between the two groups (Figure 2B,D). Immunostaining of the proliferation-marker Ki67 in 4T1 tumors showed no significant difference in the amount of proliferating cells in carcinomas obtained from β3-KO or WT mice (Figure 3). This was expected due to similar tumor growth rate.

**Figure 4:**
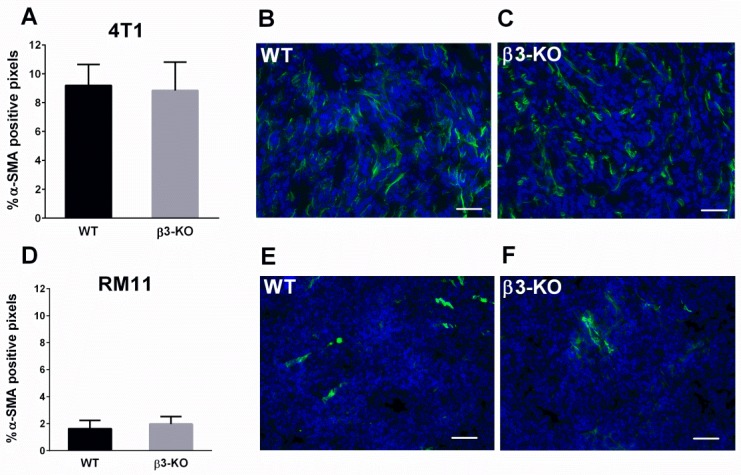
Percentage of pixels positive for α-SMA in 4T1 (*n* = 5) and RM11 (*n* = 4) tumors (**A**,**D**) from WT and β3-KO mice were calculated from immunofluorescent images. No statistical differences in 4T1 (*p* = 0.76) or RM11 (*p* = 0.34) tumors were found. Mean ± SD. Representative images of α-SMA-staining (green) from both genotypes in 4T1 (**B**,**C**) and RM11 (**E**,**F**) tumors are shown. Scale bars indicate 50 µm.

α-SMA immunofluoresscent stained tumor sections were used to quantify the relative amount of activated fibroblasts in the tumors. There were no differences in expression of α-SMA in 4T1 (*n* = 5) (Figure 4A–C) or in RM11 tumors (*n* = 4) (Figure 4D–F) between β3-KO compared to WT mice.

**Figure 5:**
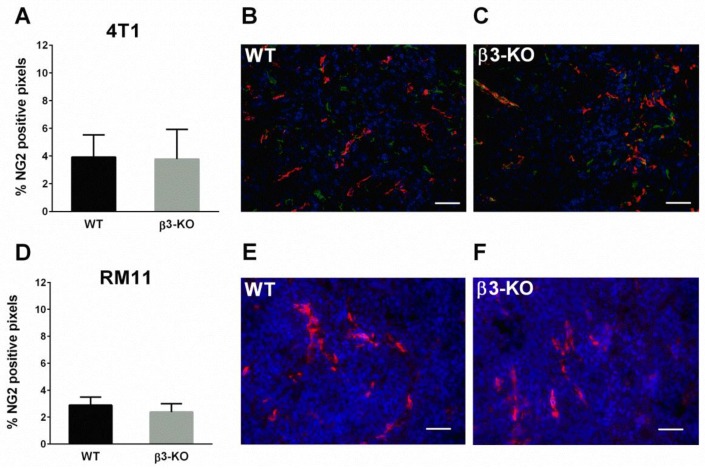
Percentage of pixels positive for NG2 in 4T1 (*n* = 5) and RM11 (*n* = 4 and *n* = 6) tumors (**A**,**D**) from WT and β3-KO mice were calculated using immunofluorescent images. No statistical differences in 4T1 (*p* = 0.90) or RM11 (*p* = 0.23) were found. Mean ± SD. Representative images of NG2-staining from both genotypes of 4T1 (**B**,**C**) (NG2 green, CD31 red) and RM11 (**E**,**F**) (NG2 red) tumors are shown. Scale bars indicate 50 µm.

Blood vessels in tumors have fewer and more abnormal pericytes than in normal tissue, and the pericyte receptor NG2 was determined as a measure of pericytes in the vasculature of the tumors. Quantification of NG2 did not demonstrate any differences in expression in 4T1 (*n* = 5) (Figure 5A–C) or RM11 tumors (*n* = 4 and *n* = 6) (Figure 5D–F) in β3-KO and WT mice (*p* > 0.05).

### 3.3. Integrin β3-Deficiency in Stromal Cells Elevates Interstitial Fluid Pressure (PIF) Only in 4T1 Carcinomas

**Figure 6:**
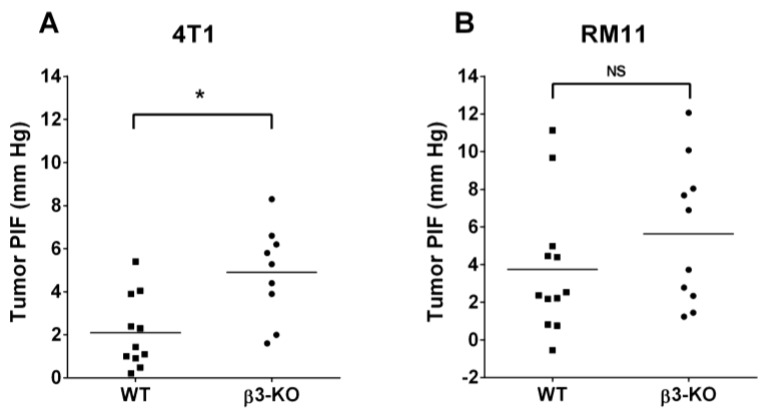
Interstitial fluid pressure (PIF) in individual 4T1 (**A**) and RM11 tumors (**B**) in WT and β3-KO mice. * *p* < 0.005, NS indicates no statistical significance (*p* = 0.24).

PIF is known to be increased in tumors and in the present study we wanted to evaluate the effect of stromal integrin β3-deficiency on PIF in tumors. The PIF measured by the wick-in-needle (WIN) technique was significantly (*p* < 0.005) higher in 4T1 tumors in β3-KO mice (4.9 ± 2.2 mmHg, *n* = 9) compared to in WT mice (2.1 ± 1.7 mmHg, *n* = 11) (*p* < 0.05) (Figure 6A). However, there was no significant difference in PIF in RM11 tumors between the β3-KO mice (5.6 ± 3.8 mmHg, *n* = 10) and the WT mice (3.7 ± 3.5 mmHg, *n* = 12) (Figure 6B).

### 3.4. Integrin β3-Deficiency in Stromal Cells Changes Collagen Architecture in 4T1 Carcinomas

**Figure 7:**
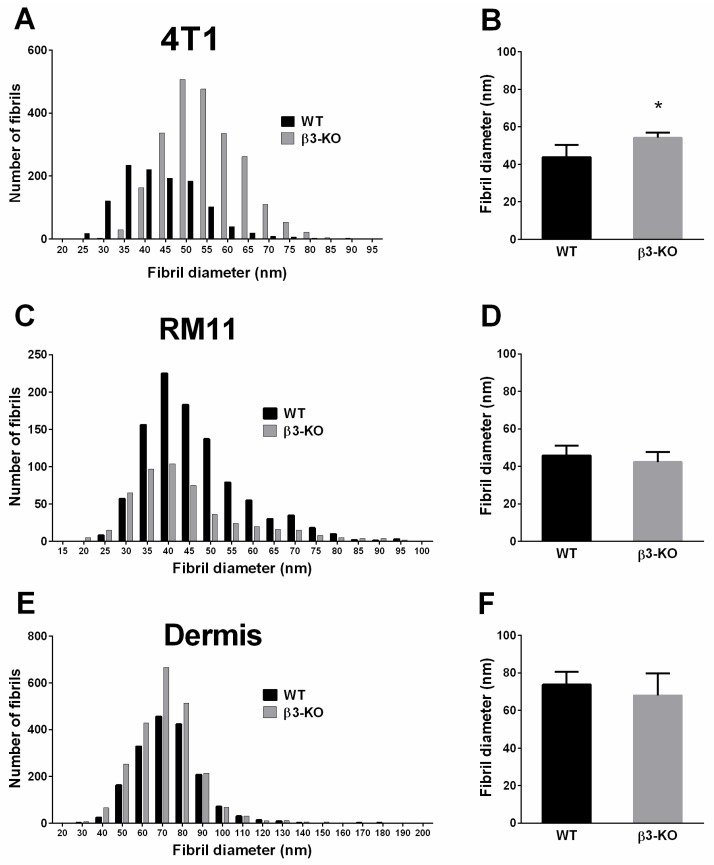
Collagen fibrils were analyzed using transmission electron microscopy. Collagen fibril diameter distribution, and average fibril diameter per tumor in 4T1 (*n* = 5) tumors (**A**,**B**), showed a shift towards thicker fibrils in KO mice. RM11 tumors (*n* = 5 and *n* = 3) (**C**,**D**) and dermis (*n* = 4 and *n* = 5) (**E**,**F**) showed no significant differences in average collagen fibril diameter in WT and β3-KO mice (RM11 *p* = 0.39, dermis *p* = 0.41). Mean ± SD. * *p* < 0.02.

Since it has been suggested earlier that collagen structure could influence PIF [32,33], we also decided to evaluate collagen structure in this study. The collagen fibril diameter from Transmission Electron Microscope (TEM) analyses of 4T1 tumors revealed an uneven distribution in fibril diameter leading to a clear shift towards thicker collagen fibrils in carcinomas grown in β3-KO mice compared to WT mice (Figure 7A). The mean collagen fibril diameter in the 4T1 tumors in β3-KO mice was significantly larger (*p* < 0.02) (54.2 ± 2.7 nm, *n* = 5) than in WT mice (43.8 ± 6.5 nm, *n* = 5) (Figure 7B). However, in RM11 tumors the mean collagen fibril diameter in β3-KO (42.3 ± 5.4, *n* = 3) was similar to that in WT mice (45.8, ± 5.2, *n* = 5) (Figure 7D).

To evaluate whether this effect on the collagen network was a specific effect on tumors grown in integrin β3-KO mice, the diameter of the collagen fibrils in dermis was also measured. Integrin β3-deficiency had no effect on the collagen fibril diameter in dermis when comparing β3-KO mice (68.0 ± 11.7 nm, *n* = 5 mice) with WT mice (73.8 ± 6.8 nm, *n* = 4 mice) (Figure 7E,F), indicating that the lack of stromal β3 integrin can specifically influence tumor fibrosis.

**Figure 8:**
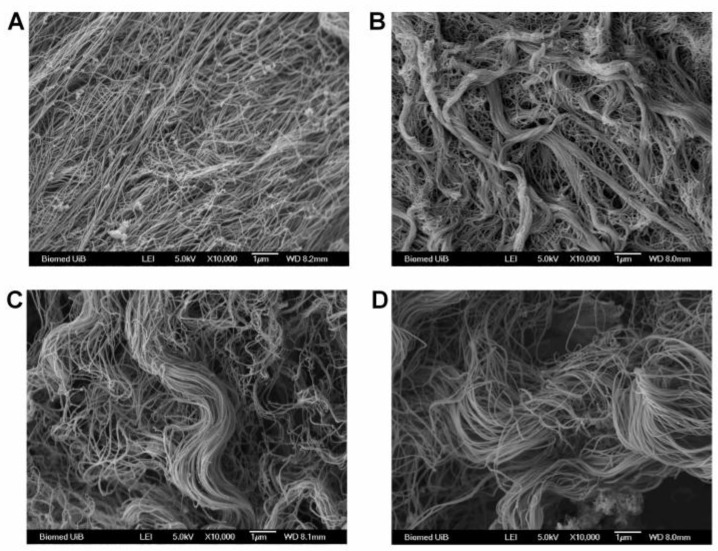
A representative scanning electron micrograph of collagen in 4T1 tumors (*n* = 6) from WT (**A**) and β3-KO mice (**B**) and RM11 tumors (*n* = 4) from WT (**C**) and β3-KO mice (**D**), respectively. Scale bars indicate 1 µm.

By using Scanning Electron Microscopy (SEM) to visualize the collagen architecture, a trend toward a thicker and denser network of collagen fibrils in 4T1 tumors (*n* = 6) in β3-KO mice compared to WT mice was observed (Figure 8A,B). This was not observed in the RM11 tumors (*n* = 4) when comparing tumors grown in β3-KO and WT mice (Figure 8C,D).

### 3.5. Integrin β3-Deficiency in Stromal Cells Does not Influence Metastasis

**Figure 9:**
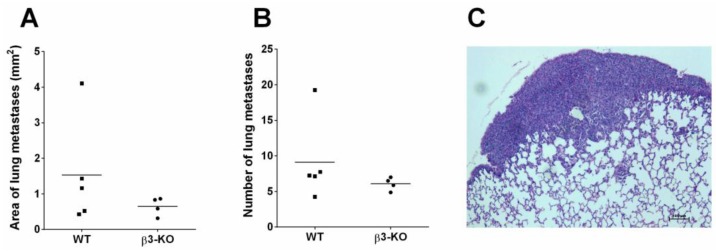
Histomorphometric quantification of H & E-stained lungs from the 4T1 model in WT (*n* = 5) and β3-KO (*n* = 4) mice. Average area per lung covered by metastases (**A**) and average number of metastases per lung (**B**) is shown. No statistical differences were found (*p* = 0.41, *p* = 0.19). A representative lung metastasis from a WT mouse is shown (**C**). Scale bar indicates 100 µm.

To evaluate whether stromal integrin β3 has an effect on metastatic potential, H & E stained sections from the 4T1 metastatic model were used. The 4T1 breast cancer cell line is known to metastasize to lungs, liver, bone and brain [34]. Excessive macroscopic surface metastases were observed in all the lungs from both β3-KO and WT mice in the 4T1 metastatic model. There was no significant difference in the ability of primary tumor cells to metastasize to the lungs in the β3-KO mice (*n* = 4) compared to the WT mice (*n* = 5) (Figure 9). No metastases were observed in the femur bones or in the livers during the time span of the 28 day study. However, in the livers the parenchyma was significantly infiltrated by isles of extramedullary hematopoiesis, thereby making it difficult to distinguish these isles from small metastases.

## 4. Discussion

The present study showed that the absence of β3 integrin in the stroma of 4T1 mammary tumors significantly elevated PIF concomitant with a shift towards thicker collagen fibrils. No change in collagen fibrils or PIF was found in the RM11 prostate carcinomas. These results support the previously suggested notion that a denser collagen scaffold will increase PIF [32,33]. Since the breast and prostate carcinomas respond differently concerning the collagen network in the stromal deficient mice, this indicates that there must be an important crosstalk between the stroma and the specific tumor cells. No major influence of stromal integrin β3-deficiency on tumor growth, angiogenesis or metastasis was found. Although initially (day 7) there was an enhanced 4T1 tumor volume in integrin β3-deficient mice, there were no significant differences in tumor volume at later time points.

The lack of a β3 integrin-effect on tumor growth corresponds to what has earlier been reported in tumor models in integrin β3-deficient mice, using CT26 colon carcinoma and LM3 breast carcinoma [32], and in a recent study by Carter *et al*. [35] where mammary tumor cells (4T1BM2 and 4T1.2) were implanted orthotopically. Several other studies on integrin β3-deficient mice, however, have shown conflicting results [36,37]. Angiogenesis is pivotal for tumor progression and the integrin αvβ3 has been proposed to have a role in this [10,21,22,38]. The present study showed no influence on tumor angiogenesis in the orthotopically implanted 4T1 or the subcutaneously implanted RM11 tumor model in the integrin β3-deficient mice, which corresponds to the lack of influence on tumor growth. This is consistent with the findings in subcutaneously implanted CT26 colon carcinomas [32] and orthotopically implanted 4T1BM2 mammary carcinomas in β3-deficient mice [35]. Nonetheless, some studies using integrin β3-deficient mice have found increased tumor angiogenesis [35,36,37], and different integrin αvβ3-inhibitiors have been shown to inhibit tumor angiogenesis in preclinical studies [21,22,23,24]. However, the present data showed no change in vessel density or morphology (NG2 staining), which is in agreement with other studies arguing against a critical role for β3 integrin in angiogenesis [32,35].

The PIF in tumors is higher than in the surrounding normal tissue and a reduction in PIF in tumors has been shown to elevate chemotherapeutic uptake and efficacy in carcinomas [39,40,41]. Several studies have shown that modulation of the collagen network has enhanced the chemotherapeutic efficacy [42,43]. Inhibition of collagen type I in carcinomas improved the intratumoral distribution and efficacy of nanotherapeutics [42], and, in a mouse model developing spontaneous pancreas tumors, it was demonstrated that reduced fibrosis enhanced the uptake and efficacy of chemotherapeutic drugs [43]. Previous studies have also suggested that fibrosis would significantly influence PIF [32,33]. Tumors grown in mice deficient in fibromodulin, a small leucine-rich protein important in organizing the collagen molecules into fibrils and fibers, resulted in lowered PIF and a loose tumor ECM [33]. This is in agreement with the present results suggesting that an elevated PIF was associated with a collagen matrix with larger collagen fibril diameter in the 4T1 tumors grown in integrin β3-deficient mice. An elevation in PIF in integrin β3-deficient mice has also previously been related to enhanced fibrosis in two different types of syngeneic murine carcinomas grown in integrin β3-subunit deficient mice [32]. Thus, our findings support the hypothesis that increased fibrosis in tumors is associated with enhanced PIF.

The magnitude of PIF is a result of capillary filtration of fluid into the tumor and drainage by lymph flow, as well as the resistance offered from the tissue between these two, *i.e.*, the hydraulic conductivity of the tissue, which is in turn determined by the matrix composition. Since blood vessel density and also pericyte density was the same in tumors in integrin β3-deficient mice and wild type mice, this suggests that the reason for the higher PIF is located in the extracellular matrix or is caused by reduced lymphatic drainage. The increased collagen fibril diameter in the 4T1 tumors is in agreement with increased hydraulic resistance (lowered conductivity) [44], however, one cannot rule out changes in lymphatic drainage as a cause for the increased PIF.

The RM11 tumors, however, did not show any change neither in the collagen network nor PIF. However, the mechanims behind elevation of PIF in tumors are not yet fully understood, so other factors might have contributed to the effect seen in the 4T1 tumors. Taken together, our findings indicate that there must be important crosstalk between the stroma and tumors, since the two different tumor models responded differently when growing in mice with identical genetic background. Thus, the effect of β3 integrin on PIF seems to be tumor cell line specific. Although, less likely, it may not be fully ruled out that the site of implantation or mice gender could play a role.

The murine 4T1 tumor cell line is known to metastasize spontaneously to lung, liver, bone, and brain via the hematogenous route [34], and the present study showed significant metastasis in all the lungs at the end point. Nevertheless, in this study we could not demonstrate any influence of stromal β3 integrin in this process since the metastatic burden was not significantly different in WT and integrin β3-deficient mice. This is in line with a study by Taverna *et al*. [45], showing no major differences in lung metastases found in integrin β3-deficient mice compared to control mice. On the other hand a study on B16 melanoma cells injected into the left cardiac ventricle has concluded that “platelet and osteoclast β3 integrins are critical for bone metastasis” [46]. It is known that integrin β3 knockout mice have defects in their platelet function, due to loss of the αIIbβ3 integrin [8], and platelets are also strongly implicated in promoting tumor progression and metastasis [47].

In a recent study, Carter *et al.* [35] reported reduced spontaneous metastasis when tumor cells with reduced β3 integrin expression were used to study metastasis *in vivo*. However, when comparing metastasis of 4T1.2 and 4T1BM2 cell lines injected into integrin β3-deficient, and wild type mice, no difference in metastatic burden was seen. They concluded that it is the tumor, rather than stromal integrin β3-expression that is essential for efficient spontaneous breast cancer metastasis to bone and soft tissue. Since integrin αvβ3-antagonists will also affect the tumor cells, this could also explain why in several studies where different integrin αvβ3-antagonists were used, it was found that these can have anti-metastatic properties [15,16,17,18].

Using a global β3-KO model there will be an issue regarding whether or not other integrins may be regulated. However, in a β3-KO C57BL/6Ntac mouse model, where the authors profiled isolated cells (platelets and MEFs) from β3-KO mice, there was no evidence for upregulation of expression of αvβ1 or αvβ5 in the β3-KO cells [8]. In another study of expression profiles and functions of integrins in WT and β3-KO endothelial cells, no changes in β5, β1, α1, α2 or α5, were found, and the authors stated that there was “no evidence for compensation by other integrins in response to β3 deficiency” [36].

The present study points to an important and complex relationship between the tumor cells and the extracellular matrix to determine PIF. The matrix and high PIF act as a functional barrier with biophysical properties for transport between blood and the tumor cells. Understanding what causes the elevated PIF and thereby how it can be decreased, may pave the way for new adjuvant therapy by allowing for enhanced transport of cytostatic agents from blood to the tumor cells. Although the present study does not clearly point to a single determinant for the elevated PIF, it points to a complex interplay between the tumor cells and the extracellular matrix.

## 5. Conclusions

The two tumor models studied here showed markedly different responses in the collagen matrix and PIF, depending on the genetic background of the mice. This strongly suggests that there is an important and likely complex crosstalk between the stroma and the specific tumor cells. Furthermore, the differences in responses in our data together with other experimental and clinical studies concerning tumor growth, angiogenesis and metastasis indicate that the basal biology of this integrin in carcinogenesis is yet not well enough understood, and more studies are needed to evaluate this further.
